# Feasibility of generating 90 Hz vibrations in remote implanted magnets

**DOI:** 10.1038/s41598-021-94240-2

**Published:** 2021-07-29

**Authors:** Jordan Montero, Francesco Clemente, Christian Cipriani

**Affiliations:** 1 The BioRobotics Institute, Scuola Superiore Sant’Anna, 56127 Pisa, Italy; 2 Department of Excellence in Robotics and AI, Scuola Superiore Sant’Anna, 56127 Pisa, Italy

**Keywords:** Biomedical engineering, Electrical and electronic engineering, Neuroscience

## Abstract

Limb amputation not only reduces the motor abilities of an individual, but also destroys afferent channels that convey essential sensory information to the brain. Significant efforts have been made in the area of upper limb prosthetics to restore sensory feedback, through the stimulation of residual sensory elements. Most of the past research focused on the replacement of tactile functions. On the other hand, the difficulties in eliciting proprioceptive sensations using either haptic or (neural) electrical stimulation, has limited researchers to rely on sensory substitution. Here we propose the myokinetic stimulation interface, that aims at restoring natural proprioceptive sensations by exploiting the so-called tendon illusion, elicited through the vibration of magnets implanted inside residual muscles. We present a prototype which exploits 12 electromagnetic coils to vibrate up to four magnets implanted in a forearm mockup. The results demonstrated that it is possible to generate highly directional and frequency-selective vibrations. The system proved capable of activating a single magnet, out of many. Hence, this interface constitutes a promising approach to restore naturally perceived proprioception after an amputation. Indeed, by implanting several magnets in independent muscles, it would be possible to restore proprioceptive sensations perceived as coming from single digits.

## Introduction

The rehabilitation of individuals that underwent upper limb amputation, using artificial replacements, still represents an important clinical issue, with suboptimal—sometimes ineffective—solutions. Interestingly, while an acceptable level of grasping function is often gained through active prostheses, restoring sensory functions remains one of the open challenges in this field.

For instance, body-powered prostheses are controlled by means of cables actuated through body movements. The user can thus infer both their aperture, from the amount of movement, and grip strength, through the reaction forces transmitted to the body by the cables^1^. Myoelectric prostheses are instead controlled by means of processed electromyographic signals recorded from residual muscles. Paradoxically, although technically more advanced than the former, they fail in providing rich sensory feedback. In fact, myoelectric hand users must rely on vision, and incidental stimuli (e.g.: audible noise from the motor or socket pressures and vibrations), to regulate their actions^2^.

Most of the past research in this area focused on the replacement of tactile functions, using different stimulation strategies. For example, vibro-tactile skin feedback has been used to signal contact events^3^, and direct electrical nerve stimulation has been employed to provide information about the grip force^4^. Hence, current technology in upper-limb prostheses, while beginning to incorporate some haptic feedback, does not provide amputees with proprioceptive information about the state of the limb.

Proprioception, or the sense of oneself, is used by the brain to track the relative position of the parts of the body, as well as their movement^5^. This information is used to control the execution of motor tasks, refining balance and movement in general. Indeed, in its absence, motor execution is prone to errors, leading to clumsy, poorly coordinated movements, inadequately adapted to complex tasks^5,6^. Besides this critical role, the congruency between intentional movements and the concurrent sensory feedback from the movements themselves provides a sense of authorship, or agency, that distinguishes one’s own actions from those of others^7^. Proprioception is intimately related to kinesthesia, which corresponds to the sense of movement of body parts^8^.

The difficulties in eliciting proprioceptive or kinesthetic sensations using either haptic stimulators or intra- or extra-neural electrical stimulation, has invited researchers in the area of limb prostheses to investigate sensory substitution as an alternative way^9–14^. While this has shed light in the domain of multi-channel sensory perception^10,13^ it has curtailed the development of a knowledge comparable to that of tactile sensory feedback^15,16^. In particular, this lack has prevented neuroscientists and neurophysiologists from investigating in a direct manner the sense of proprioception in humans, which in turn have focused on animal models^17,18^. Very recently Clites et al. proposed and tested, with a transtibial amputee, a surgical technique called agonist–antagonist myoneural interface (AMI), in which antagonist muscles are surgically connected in series so that contraction of one muscle stretches the other^19^. This architecture preserves the dynamic muscle relationships that exist within native anatomy, thereby allowing proprioceptive signals from mechanoreceptors within both muscles to be communicated to the central nervous system.

One promising approach to restore a piece of proprioception, namely kinesthesia, exploits the so-called tendon illusion. This is an illusion of movement elicited by mechanically vibrating the tendons or the muscles, through the skin, at a frequency of 70 to 100 Hz^20–22^. It is believed that such vibration excites sensory elements within the arm, which convey to the brain information about the perceived displacement and speed of the joint^5^. In their work, Marasco and colleagues have demonstrated that the addition of kinesthetic feedback improves the control of upper-limb prostheses, when compared to the vision-only control case, in people with targeted muscle and sensory reinnervation^23^.

In this work, we merged different disciplines in order to propose a new concept for a system potentially able to restore kinesthetic feedback from the inside of the muscles, taking advantage of the tendon illusion. It consists of a number of miniature permanent magnets, to be implanted in the residual muscles following amputation, that are finely vibrated by a number of magnetic field sources placed externally. In its foreseen implementation, pairs of magnets are implanted in antagonist muscle pairs, and are individually vibrated, according to sensors in the prosthesis, in order to stimulate the sensory elements contained within individual muscles. Hence, this approach, termed myokinetic stimulation interface, could partially restore the patient’s proprioceptive sense of the missing extremity. Notably if such interface is coupled with a system capable of localizing the position of the implanted magnets, and of controlling the movements of the prosthesis^24,25^, the whole system could restore the natural sensorimotor control loop.

Vibration (or more generally, steering) of a remote magnet is possible by means of the interaction of its magnetic field with the one produced by external magnetic sources. These can be either moving permanent magnets or electromagnetic coils flown by static or low-frequency currents^26,27^. The use of the latter is preferred, because it yields simpler and more compact systems when dealing with the actuation of multiple magnets, as demonstrated in a number of works^28–31^. One of the first medical applications of magnetic steering was remote catheter guidance, achieved through coil arrays^32,33^. More recent medical applications include magnetically driven endoscopes^34,35^ and drug delivery systems^36^.

Brewer and colleagues^37^ proposed a system for minimally invasive surgery, based on an external driving magnet, and the use of movable magnetic shields. The dimensions of such a setup were considerably smaller than the aforementioned ones. The system, although simple, allowed to control only the linear movement, along one dimension, of a single magnet. Wong et al. used coils wrapped around the ends of a tube to implement one-dimensional motion control of multiple magnetic micro-robots inside the tube^38^. In an extension of their work, they used an array of four coils to control the 2D forces applied to two mm-sized robots, using the visual feedback captured by one camera^29^. Chowdhury and team followed a similar approach, to control the motion of two microrobots along a plane, while avoiding obstacles^30^.

Kummer proposed a system based on eight coils, aptly called “OctoMag”, to control the five degrees of freedom (DoF) of a surgical microrobot in a tridimensional space^39^. The Octomag system was able to accurately steer the microrobot inside an oil suspension, and to use it to puncture the blood vessels of a chicken embryo. This procedure simulated the puncture of a human retinal vessel. The 3D steering of multiple micro-robots was further explored by Diller and coworkers, using rotating magnetic fields produced by an array of coils^28^. Their work established the minimum conditions required to independently control all the DoFs of a set of microrobots. They also gave insight into how to reduce this number by using a rotating field, and by exploiting the physical properties of each robot. Other examples of techniques used for magnetic steering include arrays of external, rotating magnets^40^ or combinations of rotating and static fields^31^, among others^26,27,41^.

All in all, while past research focused on the remote control of slowly moving magnets, the control of movements with large dynamics, such as vibrations, received little attention. Moreover, since past applications did not, in general, require a fast response time, issues concerning real-time operation have been overlooked. The use of optimization algorithms and numerical solvers^28,31^ for the calculation of coil currents might be, for instance, problematic for a high-speed real time implementation.

This work, thus, investigates the viability and the real-time implementation of a system able to selectively vibrate a number of permanent magnets suspended in a material resembling the viscoelastic properties of the human forearm muscles, within a tridimensional workspace. The system included 12 coils placed around the workspace (that mimicked the dimensions of a 99th percentile male human forearm^42^), which controlled in a feedforward fashion the magnetic field in order to induce torsional or linear vibrations in four magnets. The magnets were actuated, along different directions, using sinusoidal reference signals. Their motion was assessed using a high-speed camera and custom image processing algorithms, in order to determine the actual vibration amplitude and frequency.

The results indicated that it is indeed possible to vibrate individual magnets along five different directions, with high directionality, especially at low frequencies. The system was able to selectively vibrate a single magnet, out of a total of two or four magnets. The best performance was obtained when torsional vibrations were produced, which showed an efficiency equal to 0.9 ± 0.1 (average ± standard deviation) across all tests. On the contrary, linear vibrations exhibited values ranging from 0.32 ± 0.18 to 0.93 ± 0.02. These results, however, varied significantly depending on the test conditions, and were significantly affected when high-frequency vibrations were generated, and when more magnets were controlled.

### Architecture of a myokinetic stimulation interface

**Figure 1:**
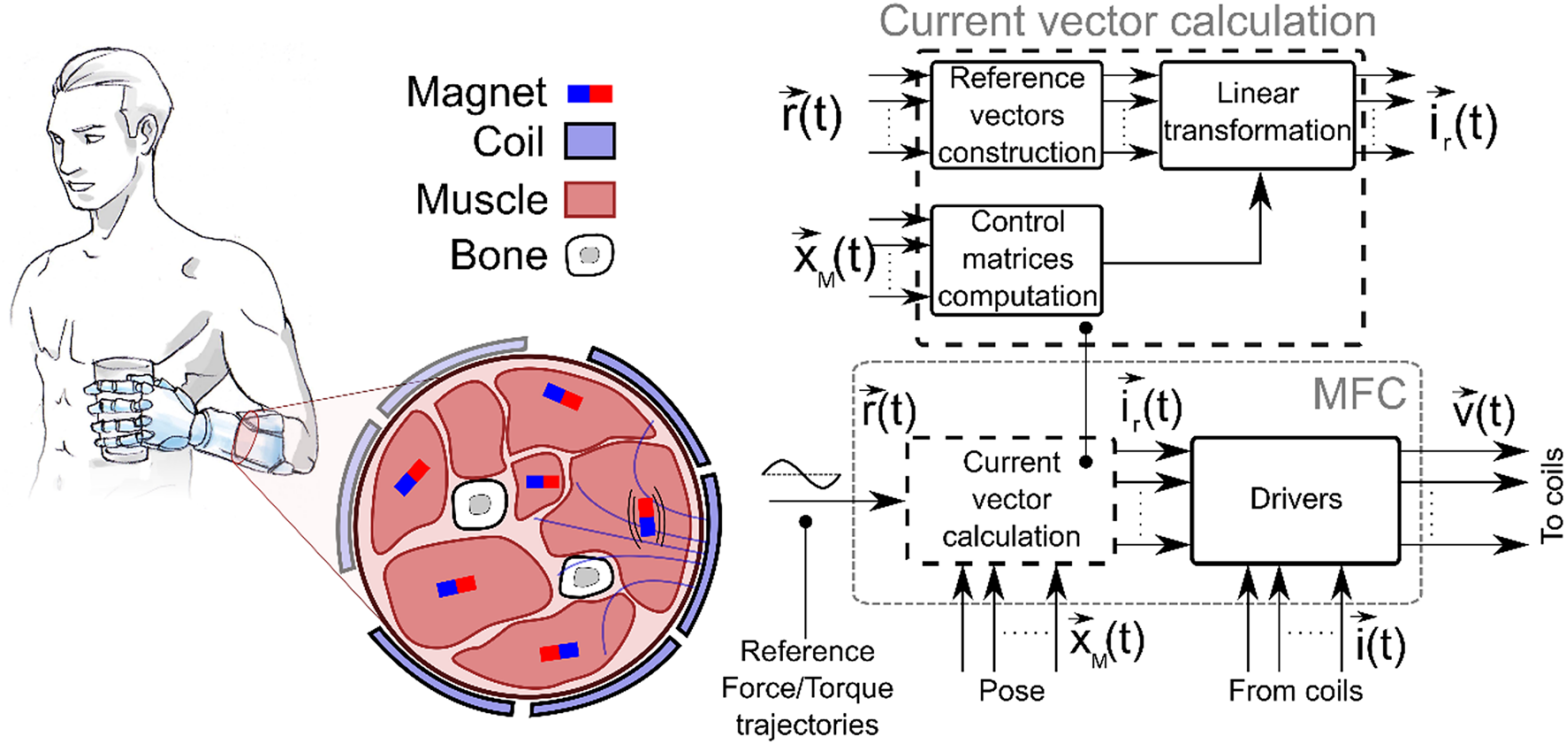
General scheme of the myokinetic stimulation interface. The system is composed of magnets implanted in the residual muscles, external electromagnetic coils, and a magnetic field controller (MFC). The MFC comprises a Current vector calculation block and a set of electrical drivers. The former is used to drive the latter. Specifically, it transforms the reference force/torque trajectories (i.e. the desired movements of the magnets) into reference currents provided to the coils which, in turn, generate the required magnetic field. In the current implementation, the position and orientation (pose) of the magnets is supposed to be known a priori.

A myokinetic stimulation interface comprises: (i) a number of magnets implanted within the muscles, with known or measured magnetic moment, position and orientation (i.e., pose), (ii) a number of electromagnetic coils distributed around the muscles, and (iii) a control system that regulates the currents in the coils in order to generate the magnetic field that produces the desired mechanical vibrations in the magnets (Fig. 1). We called such control system the magnetic field controller (MFC). It generates vibrations in the 70–100 Hz range, which are known to elicit kinesthetic percepts^20–22^.

In the present form, the MFC can be divided into a computational and a physical part, respectively, a current vector calculation block, and a bank of electrical drivers for the coils (Fig. 1). The former computes, at each time step, the vector of currents, $\vec{i}_{r}$, as well as the transformation matrices $\overline{\overline{M}}_{f}$, $\overline{\overline{M}}_{\tau }$, $\overline{\overline{M}}$, and $\overline{\overline{M}}^{ - 1}$ (Eq. (12) in supplementary information) based on the desired magnetic field. To do so the poses of the magnets should be known or measured at each time step. In the present implementation the matrices were computed only once at *t* = 0, considering that only negligible displacements were involved. The drivers deliver the current $\vec{i}_{r}$ to the coils.

**Figure 2:**
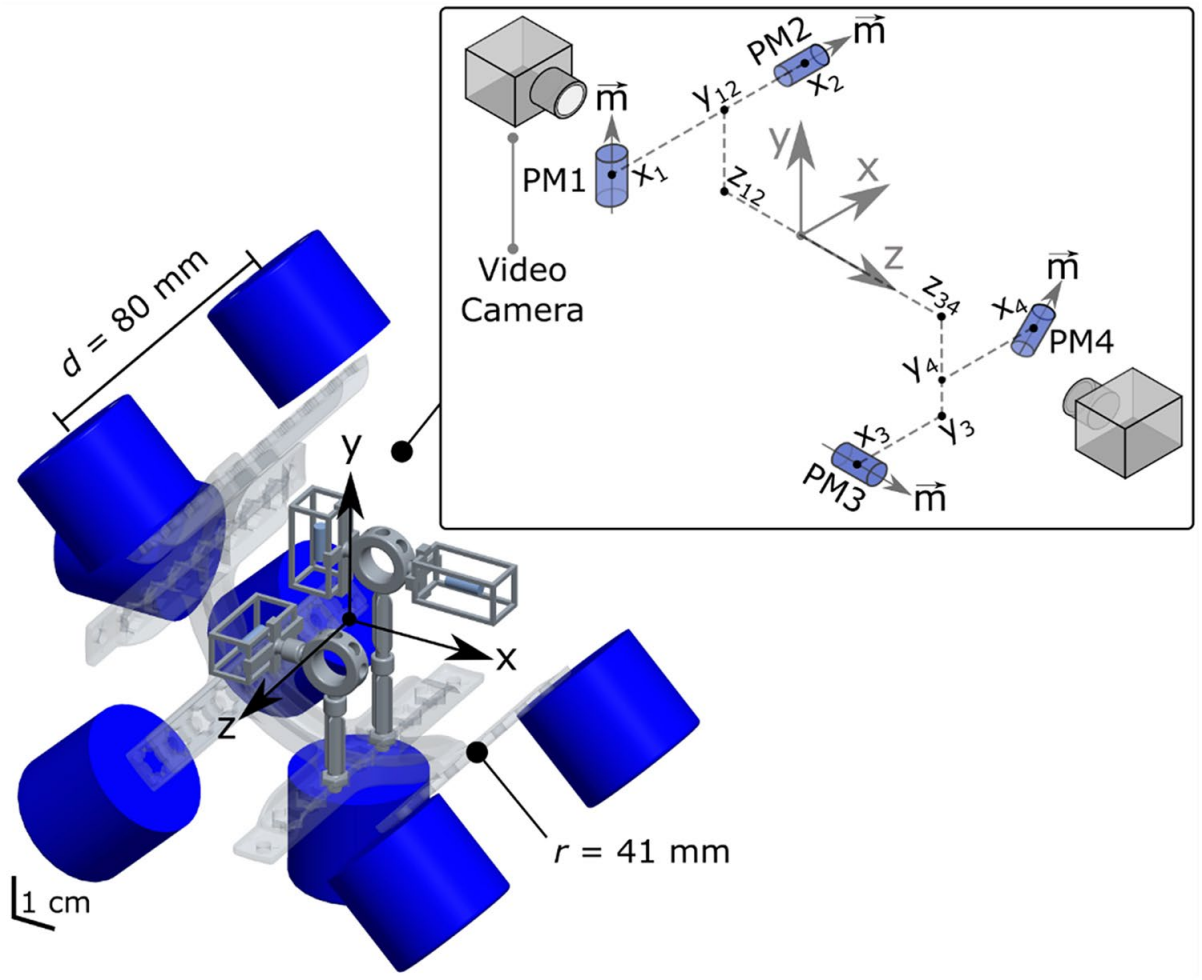
Prototype of the myokinetic stimulation interface and related characterization setup. The prototype (only half shown for the sake of clarity) consisted of a 3D printed non-magnetic cylindrical frame (*r* = 41 mm), simulating the human forearm, and twelve coils, uniformly distributed around three parallel rings (axial distance between rings *l* = *d/*2 = 40 mm), were fixed on the frame outer surface so to point towards its axis. Four magnets (PM1-PM4, only three shown) were suspended inside gelatin capsules (not shown) inside the frame. Inset: spatial configuration of the magnets during the assessment. PM2, PM1 and PM3 were oriented parallel to axes *x*, *y* and *z*, respectively. PM4 was rotated by 45° in the *xy* plane. During the tests, a camera was placed in one of the two indicated positions, depending on the PM being observed.

In the present implementation, 12 coils were used. The latter could theoretically implement a control over 12 degrees of freedom (DoFs), i.e., for example, full control over two permanent magnets (3 DoFs of force and 3 DoFs of torque, for each magnet), or a partial control over three or more magnets (cf. Supplementary Text). In this work, four magnets were used. The chosen electromagnets were commercial components (ITS-MS-5030-12VDC, produced by Intertec Components GmbH, Germany) consisting of a coil embedded in a cylindrical ferromagnetic case (*d* = 50 mm; *h* = 30 mm). As for the magnets, we employed commercial cylindrical NdFeB permanent magnets (*d* = 3 mm; *h* = 8 mm; axial N48 magnetization). The coils were distributed, as three parallel rings, around the outer surface of a cylinder (*r* = 41 mm) that simulated a human forearm (Fig. 2). The four magnets were placed inside the cylinder, instead. Three of them were aligned with the three orthogonal axes composing the reference frame. One magnet was tilted, and lied in a plane perpendicular to the axis of the cylinder (Fig. 2).

The MFC was implemented as a C++ application, executed on a PC running a RTOS (Ubuntu Linux with the RT-PREEMPT patch, 500 Hz control frequency), complemented by commercial motion controllers used as drivers (MC5004 P RS/CO on a MC5004 MB4 motherboard, from Faulhaber Minimotor SA).

A signal generator was also implemented within the C++ application to test the MFC by providing reference sinusoidal force/torque trajectories. In the present implementation the desired signal frequency and amplitude could be chosen manually. In the eventual clinical implementation these signals would be provided or modulated by sensors in the prosthesis, e.g. movement of a digit, to provide the desired feedback to the patient.

## Results

### Characterization of the MFC

Each magnet and coil was modelled after the magnetic dipole model. By doing so, only their magnetic moment was needed to calculate $\overline{\overline{M}}$. These were thus experimentally calculated for a population of coils and magnets. This was performed by mapping the magnetic field generated by each magnetic source and fitting the dipole model to the measurements.

The magnetic moment of the coils magnetic field, *m*_*c*_, was found equal to 1.531 ± 0.059 Am^2^ (average ± standard deviation, *C*  = 12), positioned at *z*_*d*_ =  − 5.726 ± 0.476 mm (reference centred on the coil flat surface). The magnets exhibited an average magnetic moment *m* of 66.26 ± 3.95 mAm^2^ (*N* = 10). For all samples, the *r*^2^ (coefficient of determination) for the fitted dipole model were always larger than 0.98, when the distance between the sensors and the center of the magnets was equal to or greater than 3 cm.

The dynamic behavior of the coils was also modelled (RL circuit) by assessing the current response to a voltage step input signal. The coils demonstrated a resistance equal to 20.0417 ± 0.1251 Ω and an inductance equal to 159.575 ± 2.7890 mH as experimentally assessed by the step response. This pair introduces a pole at approximately 20 Hz.

**Figure 3:**
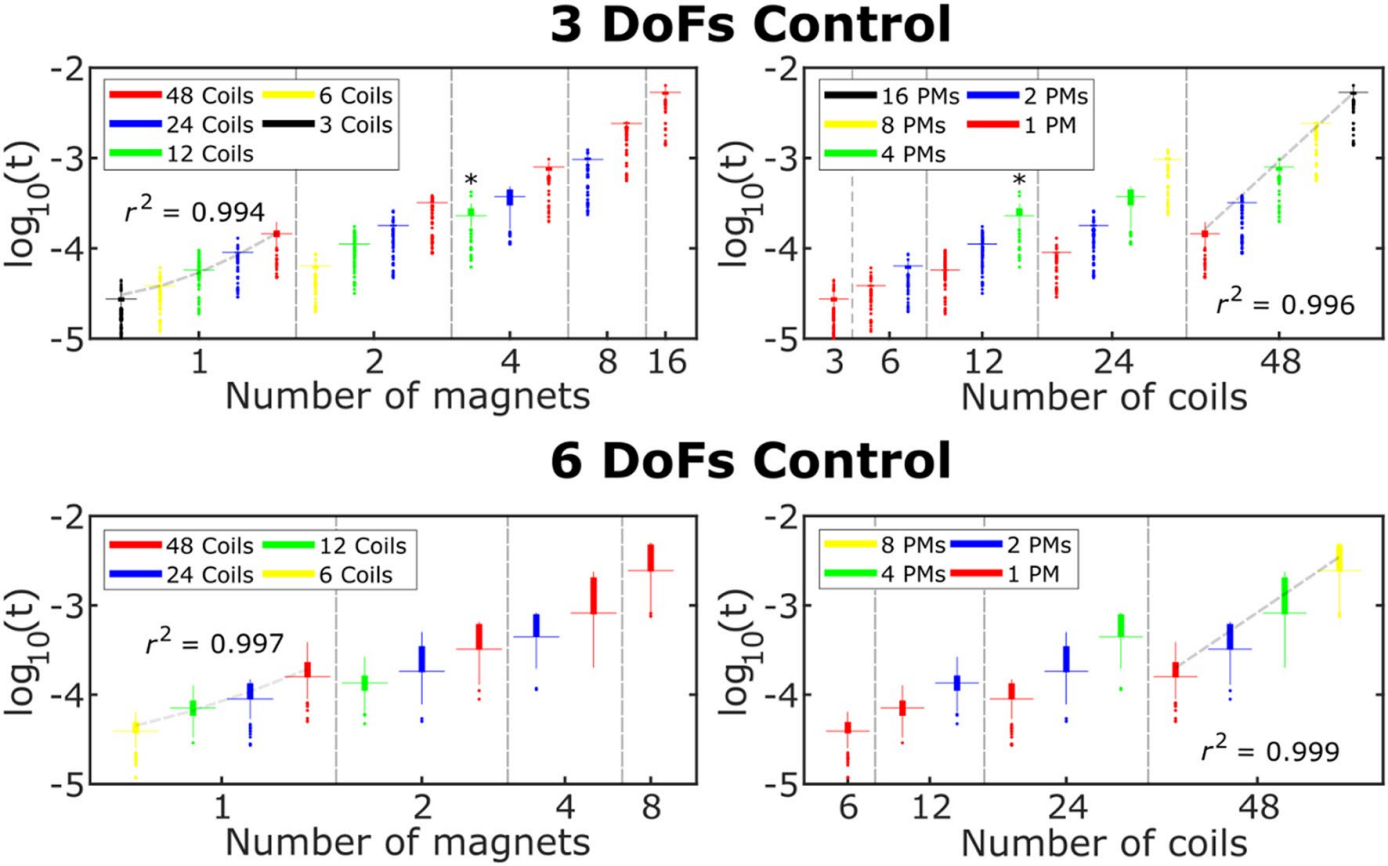
Control matrices computation time. The distribution of the computation time as a function of the number of coils with fixed magnets (left panels) or magnets with fixed coils (right panels), for the 3-DoF (force or torque) and 6-DoF (force and torque) control. The left plots were fitted using a linear function (*t(N)* = *mN* + *b*, *m* = 23.442 ± 0.991). The plots on the right were fitted using a power function (*t(C)* = *kC*^*p*^, *p* = 1.314 ± 0.087). The coefficient of determination (*r*^2^) is indicated for some representative cases. *Indicates the implemented configuration.

Finally, the time required to compute and invert the control matrices ($\overline{\overline{M}}_{f}$, $\overline{\overline{M}}_{\tau }$, $\overline{\overline{M}}$, and $\overline{\overline{M}}^{ - 1}$) was characterized, through stopwatch functions in the C++ application, as a function of the number of magnets (*N*) and coils (*C*) exploited by the MFC. This time ranged from ~ 27 ms for controlling the force on one magnet using three coils, to 2.46 ± 1.2 ms for controlling the forces and torques over eight magnets using 48 coils (Fig. 3). Not surprisingly, the time required to compute the control matrices increased with the dimensionality of the problem, i.e. with the number of coils, magnets or controlled variables. In particular, the time increased as a power of the number of magnets, when the number of coils was kept constant, while it increased linearly with the coils when the number of magnets was fixed. For the implemented configuration (*N* = 4, *C* = 12), the computation time was ~ 230 ms (Fig. 3).

### Assessment of the remote vibrations

The ability of the myokinetic stimulation interface in generating selective (in terms of direction and frequency) vibrations was tested by applying low (1 Hz) and high (90 Hz) frequency sinusoidal input signals to the MFC. The temporal evolution of the resulting position and orientation (i.e. pose) of the controlled magnet(s) was analyzed, through spectral analysis, to compute efficiency coefficients (*η* for linear vibrations, *γ* for torsional vibrations). Specifically, the maximum efficiency is obtained when the vibration is generated at the selected frequency and along the specified direction only (see the Materials and Methods section for details). Additionally, the undesired (i.e. along other directions) torsional and linear displacements were computed for the moved magnet (*θ* and *ρ*, respectively) and still magnets (*θ*_*all*_ and *ρ*_*all*_, respectively), i.e. magnets forced to not move by the MFC, if any. Tests were separated in five different groups, which varied in the number of controlled magnets (Groups I and II: 1, Group III: 2, Group IV: 4, Group V: 3) and the number of controlled DoFs (3- or 6-DoF per magnet, i.e. force and/or torque control). Several repetitions were performed by changing the set of involved magnets. In all cases, the uncontrolled magnets were not included in the analysis.

### Groups I and II: single magnet 3-DoF and 6-DoF control

**Figure 4:**
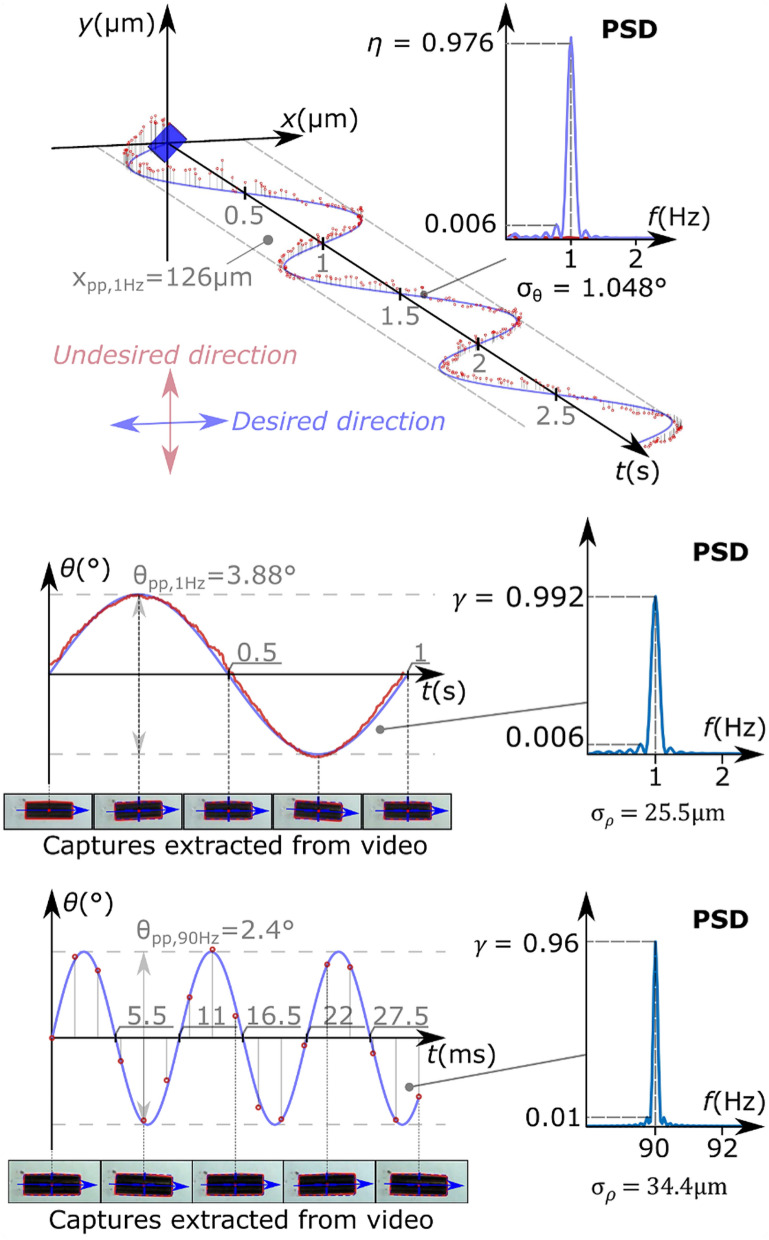
Actuation of a single magnet for three representative configurations (3-DoF control tests). The temporal evolution of three different vibration patterns and their corresponding (normalized with respect to the total power) power spectral densities (PSD, insets) are presented. Above: low-frequency (1 Hz) linear vibration along *x* of PM4. The initial orientation of the magnet is presented at *t* = 0. Middle/Below: low/high-frequency torsional vibration along *z*, of PM2. The blue waveform indicates the desired magnet trajectory. The red circles (or traces) indicate the actual trajectory, as extracted from the video analysis. *σ*_*θ*_/*σ*_*ρ*_ indicate the standard deviation of the torsional/linear displacement and *η*/*γ* the corresponding efficiency.

The several tests demonstrated that it was possible to produce, in single magnets, highly directional and frequency selective vibrations along different axes, using a 3-DoF control. For example, *η* proved equal to 0.976 for PM4 along *x* under 3-DoF force control at 1 Hz (Fig. 4, upper inset). The standard deviation of the torsional displacement (*σ*_*θ*_), proved rather low, being equal to 1.05°. Examples of 3-DoF torque control over PM2 showed *γ* equal to 0.992 at 1 Hz (i.e. LF condition), slightly reduced to 0.96 at 90 Hz (i.e. HF condition); the standard deviation of the linear displacement, *σ*_*ρ*_, followed the same trend, being as low as 25.5 mm and 34.4 mm, respectively.

**Figure 5:**
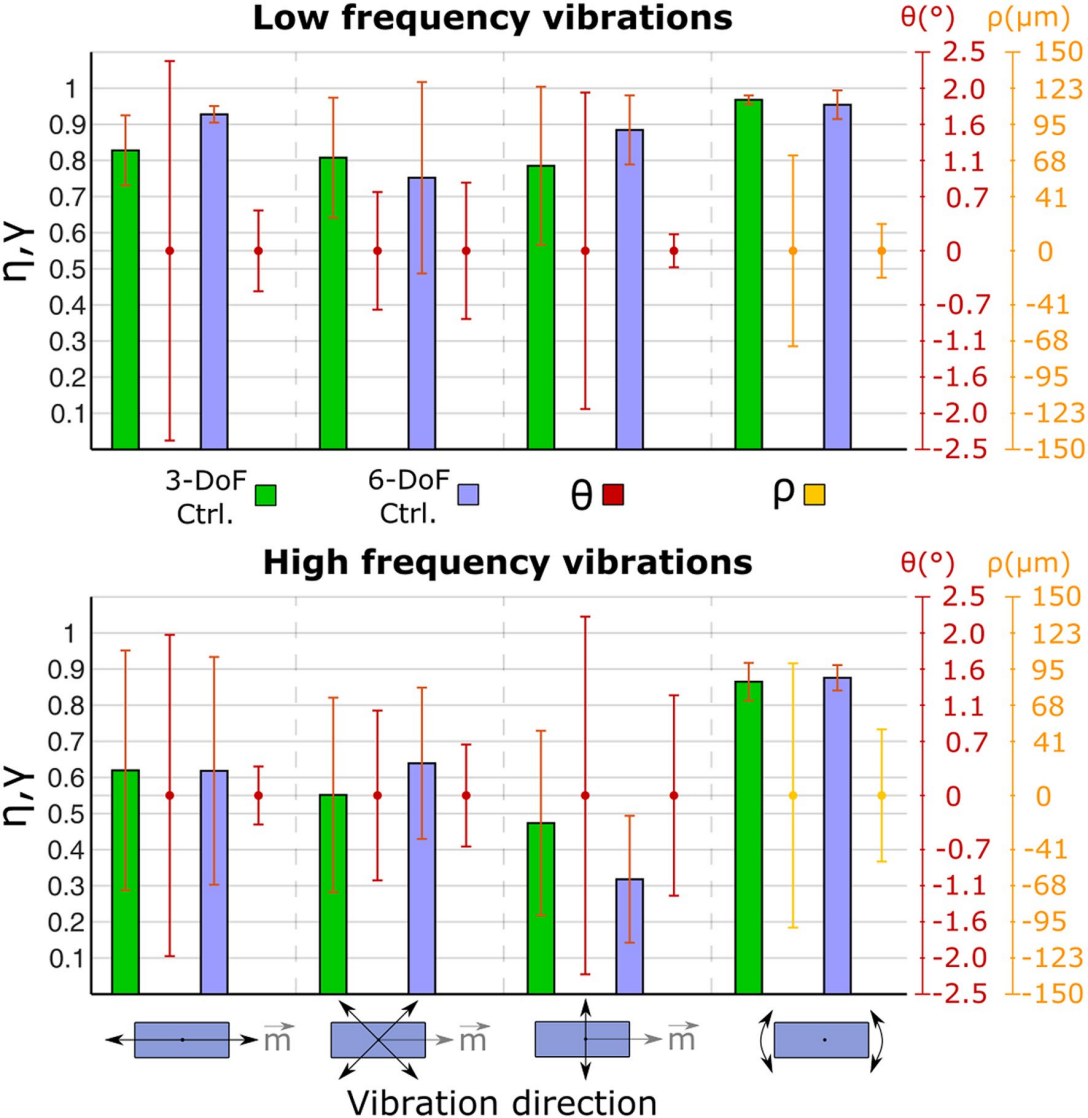
Efficiency and deviation for the single magnet 3- and 6-DoF control tests (Groups I and II). The actuation direction with respect to the orientation of the magnets is indicated with black arrows. Bar plots (mean ± standard deviation) indicate the efficiency (*η*, *γ*) of the generated movements (3-DoF in green, 6-DoF in blue). The dots (mean ± standard deviation) indicate the displacement signal (*θ* or *ρ*, depending on the test condition).

The high efficiency under 3-DoF control proved a general trend across most low-frequency tests (Fig. 5, upper panel). *η* ranged from 0.79 ± 0.22 (average ± std. dev.), for vibrations perpendicular to $\vec{m}$, to 0.83 ± 0.10 for vibrations along $\vec{m}$. *γ* was equal to 0.97 ± 0.01. When the actuation frequency was 90 Hz (Fig. 5, lower panel), both efficiencies (i.e. *η* and *γ*) decreased, although the impact on *γ* was lower (i.e. reduced to 0.86 ± 0.05). The displacements, and most notably, the linear displacement (*ρ*), exhibited a standard deviation larger at 90 Hz than 1 Hz (doubling in some conditions—not shown). When the 6-DoF control was employed (Fig. 5), the average efficiency increased in most cases (both at LF and HF), although not considerably. Additionally, the spread of the efficiency was usually lower (i.e. they exhibited a lower standard deviation), indicating a higher consistency across all test conditions. The deviations were also lower w.r.t. 3-DoF control, and less spread, in almost all cases. In all the tested conditions, the mean of the displacement distributions (*θ* and *ρ*) was approximately zero.

### Group III: two magnets 12-DoF (6–6) control

**Figure 6:**
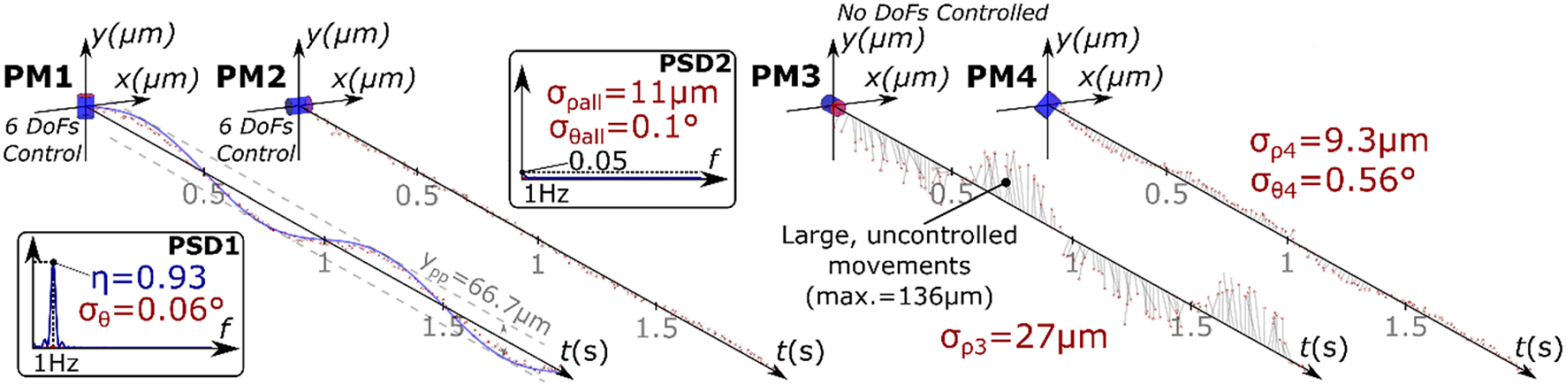
Temporal evolution of the poses of all magnets in a representative 12-DoF control configuration (Group III). A 6-DoF control was employed for PM1 and PM2. PM1 was vibrated along *y* at 1 Hz, and PM2 was kept still. PM3 and PM4 were left uncontrolled by the MFC. The initial orientation of each magnet is presented at *t* = 0. The blue line indicates the desired magnet trajectory. The red circles indicate the actual trajectory, as extracted from the video analysis. *σ*_*θ*_/*σ*_*ρ*_ indicate the standard deviation of the torsional/linear displacement and *η*/*γ* the corresponding efficiency. Insets: power spectral density of PM1 (PSD1) and PM2 (PSD2).

The 12-DoF tests also proved the possibility of generating vibrations, in any desired magnet, while keeping fixed any other one. For instance, we were able to selectively vibrate PM1 at 1 Hz, with high directionality (*η* = 0.93, *σ*_*θ*_ = 0.06º), while keeping PM2 almost still ($\sigma_{{\rho_{all} }} \approx 8$ μm, $\sigma_{{\theta_{all} }} = 0.1^\circ$) (Fig. 6). Indeed, the power spectrum of PM2 pose signal (PSD2 in Fig. 6) revealed very low signal power (~ 0.01 in the normalized PSD) at the frequency of interest (1 Hz). On the contrary, the magnets that were disregarded by the MFC exhibited larger torsional ($\sigma_{{\theta_{4} }} = 0.56^\circ$) and linear displacements ($\sigma_{{\rho_{3} }} = 27$ μm, $\sigma_{{\rho_{4} }} = 9.3$ μm), with respect to the controlled magnets.

**Figure 7:**
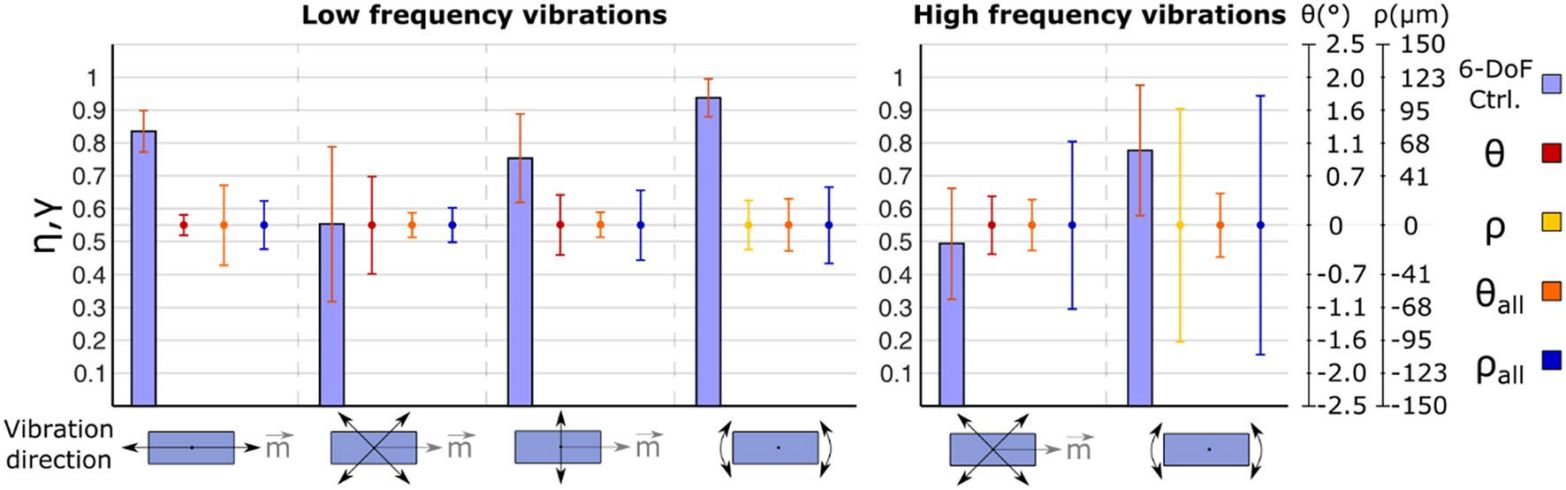
Efficiency and deviation for the two magnets 12-DoF control test (Group III). In all cases, one magnet was kept still and a second one was moved along directions indicated with black arrows. Bar plots (mean ± standard deviation) indicate the efficiency (*η*, *γ*) of the vibrations generated on the moved magnet (6-DoF control). The dots (mean ± standard deviation) indicate the displacement signal for the moved (*θ* or *ρ*, depending on the test condition) and still (*θ*_*all*_, *ρ*_*all*_) magnets.

Following the same trend of the single magnet tests, the two-magnet 12-DoF control tests resulted in a large efficiency when low-frequency vibrations were applied along $\vec{m}$ (Fig. 7, left panel), where *η* = 0.84 ± 0.06 was obtained. The best efficiency was obtained with the torsional vibrations (*γ* = 0.94 ± 0.06 at 1 Hz;  *γ*  = 0.77 ± 0.19 at 90 Hz), but they also exhibited some of the most spread distributions of the displacement signals *θ*_*all*_ and *ρ*_*all*_. Indeed, when the vibration frequency was 90 Hz, *ρ*_*all*_ proved more than 1.5 times larger for the torsional vibrations (Fig. 7, right panel). *θ*_*all*_ increased as well, but to a lesser extent. The deviations (*θ*, *θ*_*all*_, *ρ*, *ρ*_*all*_) exhibited close-to-zero mean values also in these cases.

### Group IV: four magnets 12-DoF (3–3–3–3) control

**Figure 8:**
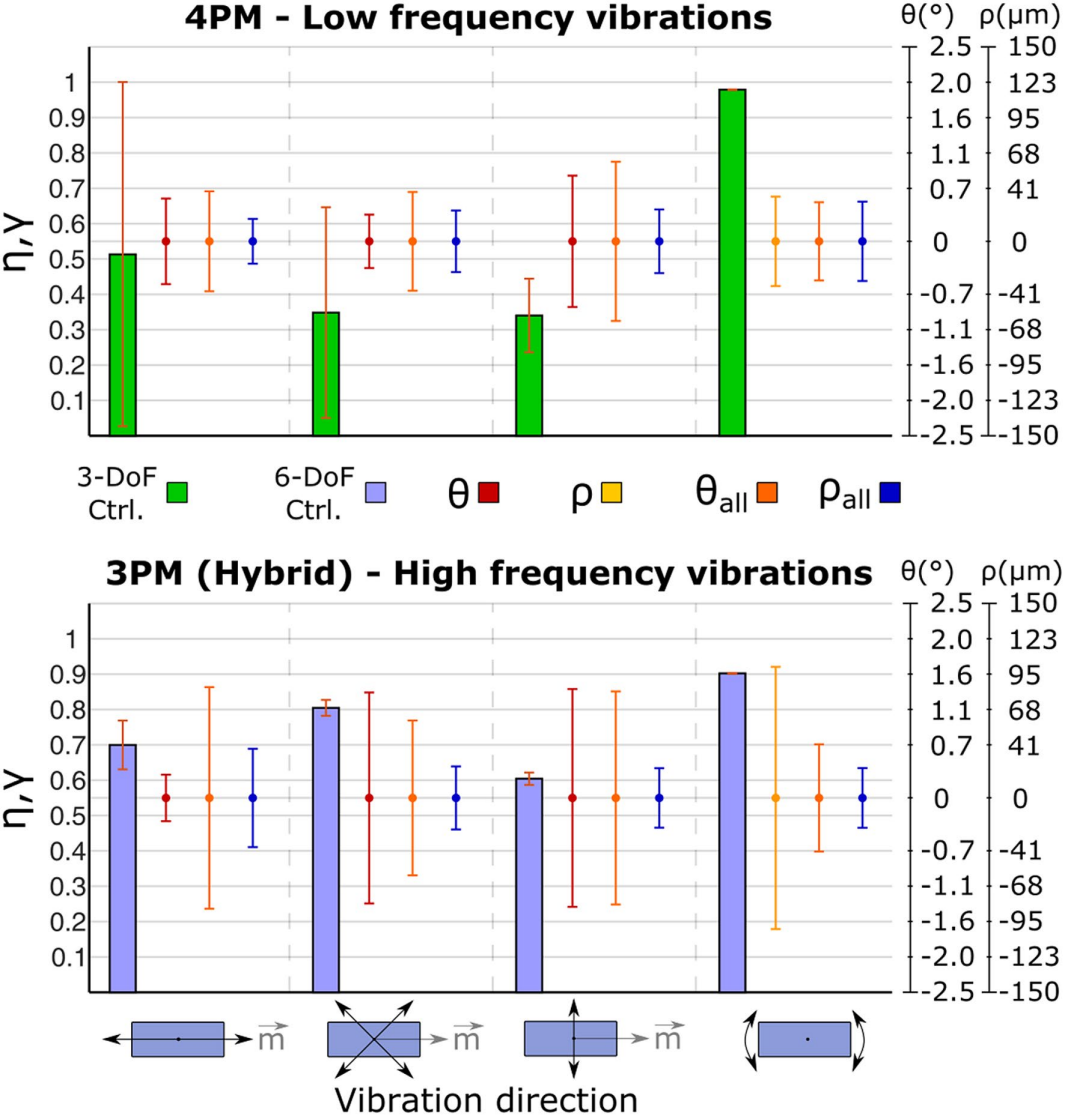
Efficiency and deviation for the four (Group IV) and three (Group V) magnets 12-DoF control tests. In all cases, one magnet was moved along directions indicated with black arrows (3-DoF control for Group IV and 6-DoF control for Group V) and all other magnets were kept still under a 3-DoF control. Bar plots (mean ± standard deviation) indicate the efficiency (*η*, *γ*) of the movements generated on the moved magnet. The dots (mean ± standard deviation) indicate the displacement signal for the moved (*θ* or *ρ*, depending on the test condition) and still (*θ*_*all*_, *ρ*_*all*_) magnets.

Not surprisingly, when the four-magnet 12-DoF control was used, there was a decrease in the efficiency (Fig. 8, upper panel) with respect to the two-magnet case (Fig. 7, left panel). Only *γ* was unaffected by the increase in the number of magnets. The distributions of the torsional displacements, *θ*, and most notably *θ*_*all*_, exhibited larger variability in almost every case.

### Group V: three magnets 12-DoF (6–3–3) “hybrid” control

The hybrid control approach (Fig. 8, lower panel) led to a slight increase of *η* and *γ* with respect to the two-magnet case (Fig. 7, right panel). The distributions of *θ* and *θ*_*all*_ increased slightly as well, but there was a considerable decrease in the values of *ρ*_*all*_, especially for the torsional vibrations. *ρ* exhibited a similar distribution in both cases. As with the previously discussed configurations, all the deviations showed a distribution with a zero-mean value (Fig. 8).

## Discussion

We presented the idea of a myokinetic stimulation interface, namely, a system able to generate remote vibrations, in implantable permanent magnets, using excitation coils. A prototype, with dimensions mimicking the human forearm, employing twelve electromagnetic coils and four permanent magnets, was implemented. The mathematical models of the magnetic components were derived, and the timing behavior of the system was assessed. In addition, several groups of tests to assess the performance of the system at generating directional and selective vibrations, were performed. These were grouped according to the following categories: vibration frequency (low, high), vibration direction (linear, torsional), number of controlled DoFs (3, 6 or 12), and number of controlled magnets (1, 2, 3 or 4).

The system proved capable of calculating the required actuation matrices (i.e. $\overline{\overline{M}}_{f}$, $\overline{\overline{M}}_{\tau }$, $\overline{\overline{M}}$), as well as the inverse of the compound matrix $\overline{\overline{M}}$, with online performance. Its inverse ($\overline{\overline{M}}^{ - 1}$) was calculated using the LUPQ decomposition^43^. The latter was chosen considering that the torque terms coming from $\overline{\overline{M}}_{\tau }$ lead to a degenerated, i.e. rank-deficient, $\overline{\overline{M}}$. Under such situation other algorithms, like the LUP decomposition, might fail^43^. Although the LUPQ is slightly more complex and computationally expensive than other algorithms (since it uses full-pivoting), with the exception of the tests with the largest number of elements (i.e. 48 coils and 8 or 16 magnets), the computation times were always below 1 ms. Remarkably, such computation time would allow the system to update the matrices at a maximum rate of 1 kHz. This is well beyond the minimum rate required to generate 90 Hz vibrations (i.e. 180 Hz as per the Nyquist theorem).

The variability of single magnet 6-DoF stimuli proved lower with respect to the corresponding 3-DoF stimuli (Fig. 5). This confirms the finding of Diller^28^ and was likely due to the large number of coils available to control the magnet (twice the required minimum number), that led to a reduction of the current applied to each coil, and to a more isotropic workspace. Nevertheless, in specific cases, the 3-DoF control yielded comparable or even better performance, as shown by a higher efficiency (*η*, *γ*). In turn, the more consistent performance across different poses exhibited with the 6-DoF control suggests that this should be preferred to the 3-DoF control in applications with a dynamic pose of the magnets. On the contrary, for those applications where the pose exhibits a limited excursion, such as in the case of the implanted magnets, a non-isotropic placement of the coils, connected to a 3-DoF control might instead be the best trade-off (reducing the number of coils).

When two or more magnets were actuated, the efficiency was generally lower than the single magnet case. This, again, was likely due to the fact that there were less coils available to control each magnet, and the workspace was thus less isotropic. For this same reason, not all actuation directions could be tested during the multiple magnets tests, since the currents required to generate movements perceivable by the video camera exceeded the current limits of the coils.

We argue that one of the reasons behind the deterioration in the performance is the lack of influence of far-away coils. For instance, when the pair PM1-PM2 was actuated, the coils closer to PM3 and PM4 had a low influence over them (Fig. 2). The same situation happened to the pair PM3-PM4, that were barely influenced by the coils closer to PM1 and PM2. For these cases, the maximum static magnetic field that far-away coils could apply to the magnets was below 500 mT (in modulus, i.e. $\vec{B}$, for *i* = 1 A). These values were up to one order of magnitude lower than those of the nearby coils, and proportionally larger currents were thus required to achieve a comparable amplitude. These larger currents led, additionally, to power-losses in the coils in the form of heat. Furthermore, due to the distance requiring unattainable currents, the mathematical model used to characterize the effect of the coils over the magnets did not accurately describe the status of the system, and the actuation performance was thus affected. We argue that this is the reason why, regardless the number of available DoFs was enough to fully control the magnets in most tests (i.e. 12 coils to control 2 magnets), the actuation exhibited poor performance in some configurations, even at low frequency (Fig. 7). Indeed, increasing the number of controlled magnets, and thus reducing the number of DoFs controlled per magnet, usually led to a decrease in the efficiency, and to larger deviations. This reinforces the notion that more coils than the required minimum should be used to ensure a higher performance.

The fact that the mean displacements were always close to zero suggests that, although undesired movements were present in many cases, there were no static (i.e. DC) components affecting the vibrations. This attests to the frequency selectivity of the system, and to the linearity of the actuation, as no undesired frequency components were generated. It also suggests that the addition of linear controllers, used to compensate the frequency characteristics of the actuators (i.e. coils), could improve the performance (i.e. the efficiency and the deviations).

Indeed, the attenuation and the lag in the current time response, induced by the dynamics of the coils at high frequencies, was significant. Hence, the currents calculated by the MFC, which were assumed to be instantaneously generated by the coils, could not be accurately followed and thus led to a deterioration in the actuation. This discrepancy between the calculated and the real currents led to an imperfect cancelation of terms in the actuation matrices, thus making the “fixed” magnet vibrate (i.e. low selectivity), as demonstrated by the larger displacements in other magnets (*θ*_*all*_, *ρ*_*all*_) during the high-frequency tests. It also led to an incorrect dimensioning of the compound magnetic fields, resulting in movements along undesired directions for the “moved” magnet (i.e. low efficiency—*η*), as it could be observed in most of the high-frequency tests involving linear vibrations. The phase shift induced in the coil currents at high frequencies generated delays in the arrival of the magnetic fields at the desired spatial positions. The resulting delayed fields could then add constructively or destructively. We argue that this is one of the reasons why the efficiency and the deviations worsened when the frequency was increased.

In general, torsional vibrations proved more efficient than linear vibrations, independently of the vibration frequency, the number of controlled DoFs, or the number of controlled magnets (Figs. 5, 7, 8). Torsional vibrations were the least affected when only the torque was controlled (i.e. when 3-DoF control was used, and the force terms were ignored), implying that more magnets could be controlled for the same number of coils. The maximum amplitude of the torsional vibrations was also considerably larger. For instance, torsional vibrations leading to displacements of up to 500 mm could be achieved, in contrast to a maximum of ~ 150 mm for the linear vibrations. One reason for this is that the force decays approximately as $\frac{1}{{r^{4} }}$ (Eq. (10)) and the torque as $\frac{1}{{r^{3} }}$ (as it is directly proportional to $\vec{B}$, as per Eq. (7)). This implies that lower currents are required to achieve larger torques. Given that lower currents are used, the modelling errors and approximations in matrices $\overline{\overline{M}}_{f}$ and $\overline{\overline{M}}_{\tau }$ have a smaller effect in the vibrations. The negative effects of the physical limitations of the actuators (i.e. saturation of the ferromagnetic material) are also diminished. Therefore, if in-vivo tests will reveal that there is no significant difference between applying linear or torsional vibrations, then using the latter would lead to a system with better performance and lower current consumption.

These outcomes suggest that the myokinetic stimulation interface can be used to activate sensory elements within the muscles in which the magnets are implanted, in real-time, and in response to stimuli provided by sensors in the prosthesis. However, it is still necessary to investigate ways of improving the performance (directionality and selectivity) of the system, especially at high frequency. The addition of closed- or open-loop controllers to compensate for the coils dynamic behavior, and to precisely control the forces and torques applied to the magnets should be considered. Closed-loop controllers based on vision systems, such as those used by Diller^28^, Chowdhury^30^, and Kummer^39^, do not constitute practical alternatives in our case, given that the implanted magnets cannot be directly observed. A system that we recently proposed^24,25^, able to retrieve the pose of multiple implanted magnets using tridimensional magnetic field sensors, might constitute a more viable choice. However, issues concerning the interaction of the coils with the sensors, and the consequent impact over the localization accuracy, will need to be assessed.

Different coil geometries, better suited to the application, should also be investigated, as they would improve the isotropy of the actuation. For instance, non-cylindrical coils, that could be embedded in the prosthesis structure, could prove particularly useful. Changing the distribution of the coils, to make them point towards a reduced actuation-region, as in the works by Diller^28^ and Kummer^39^, could improve the isotropy as well. This, however, might prove challenging in our case, given that the coils should be embedded in a structure that sits around the patient’s forearm. Adding more coils to improve the magnetic field coverage, at the expense of reducing their radial dimensions, might constitute a better choice. The use of optimization algorithms to calculate the coil currents, akin to those used by Diller^28^ and Chowdhury^30^, could also significantly improve the actuation performance of the system. These algorithms, however, would need to be adapted to operate in a low-latency, real-time setup. These topics will be the aim of future works.

To conclude, although our goal was to investigate technical and physical constraints while vibrating remote magnets, it stands to reason to project this work towards its final clinical application, by discussing a few relevant points. It is well-known that when a foreign body having the size of our magnets is implanted in a muscle, the tissue reacts by encapsulating it in a fibrotic scar, which prevents migration within the muscle^44,45^. Hence, while it is rather likely that the mechanical vibration would not damage the fibrotic capsule, this remains to be assessed. Another potential adverse effect is induction heating in the magnets; we argue that the distances between the coils and the magnets, as well as the range of usable currents, would prevent such effects. This was qualitatively confirmed during the tests described here, during which no significant increase in temperature was found in the magnets. Finally, with regards to biocompatibility and biostability issues of the implanted magnets, it is worth to report that our group recently demonstrated parylene-C covered magnets as an ideal candidate^46^.

## Materials and methods

### Characterization of the MFC

The MFC may work if the elements of the matrix $\overline{\overline{M}}$,—the magnetic moment of the magnets, *m*, and of the coils, *m*_*c*_—are known or modelled. However, off-the-shelf components, such as those used in the present system, are commonly poorly modelled and thus were experimentally characterized (Supplementary Fig. S1). This was done using a 3D Hall-effect sensor (HMC5983, Honeywell; ± 8 G range, 4.35 mG resolution) mounted on a three-axis high precision characterization bench employing three linear stages (VT-80, PI miCos).

The field produced by the coil was measured over two planes orthogonal to its flat surface, intersecting at the center of the coil, in steps of 4 mm in all directions. An area of 80 mm (horizontal direction) by 80 mm (vertical direction) was covered in each plane. The coil was fed with a DC current varying from 50 to 500 mA (in 50 mA steps), using differential measurements to cancel out the effect of common-mode components (e.g.: the geomagnetic field). 200 measurements were collected at each step (100 per polarity), and their mean value was used to build a map of the field as a function of the current and position. Symmetry was then exploited to derive a 3D model of the field and the resulting maps were fitted to Eq. (5), to estimate *m*_*c*_, and the fictitious position of its source, *z*_*d*_. Twelve coils were tested, and the average *m*_*c*_ and *z*_*d*_ were taken as the results. Whenever the sensor saturated, because the applied currents led to fields exceeding the maximum measurement range, those samples were discarded.

The field produced by the magnets was measured following a similar procedure. An area of 80 mm (horizontal direction) by 48 mm (vertical direction) was covered in each plane. 100 measurements were taken in each position. The average *m* across ten magnets was taken as the result.

The dynamic behavior of the coils was experimentally modelled by assessing the response to the step function. Voltage step inputs were applied to each coil and the current response was then measured. 100 ms of data were gathered per trial. Different voltage steps (10, 20 and 30 V) were tested (30 repetitions for each step). Values of *R* and *L* were determined, by fitting the current response to that of the RL circuit. The final values were computed by taking their mean.

The computation of matrices $\overline{\overline{M}}_{f}$, $\overline{\overline{M}}_{\tau }$, $\overline{\overline{M}}$, and $\overline{\overline{M}}^{ - 1}$ is one of the most time-consuming operations, especially due to the calculation of the inverse of $\overline{\overline{M}}$. This depends on the number of the magnets, *N*, and of the coils, *C*. The time required to calculate $\overline{\overline{M}}_{f}$, $\overline{\overline{M}}_{\tau }$, $\overline{\overline{M}}$, and $\overline{\overline{M}}^{ - 1}$ was thus characterized, as a function of *N* and *C*, using stopwatch functions in the C++ application. Although in the present setup we used 12 coils and four magnets, we measured the computation time for *C* equal to 6, 12, 24 and 48, and for *N* equal to 1, 2, 4, 8, 16, in order to draw a more general picture. In particular, for each combination of *N* and *C*, three different configurations were used to construct $\overline{\overline{M}}$, i.e.: (i) controlling the force and torque of each magnet ($C \ge 6N$), (ii) controlling only the forces ($C \ge 3N$), or (iii) only the torques ($C \ge 3N$).$\overline{\overline{M}}^{ - 1}$ was calculated using the LUPQ decomposition^43^.

### Assessment of the remote vibrations

A 3D printed prototype was developed to precisely hold the coils and the permanent magnets in known positions (Fig. 2). Four permanent magnets, PM1-PM4, were distributed in the workspace so that each was aligned on a different axis, three of them being orthogonal (Fig. 2). In particular, the coordinates were chosen to ensure that the distribution was not symmetrical (i.e., elements not lying in a circle). In turn, the largest coil to magnet (radial) distance was 8.8 cm. Each magnet was kept in place embedded inside a capsule made of ballistic gelatin (G2500, Sigma-Aldrich Corporation; 10% concentration), that simulated the viscoelastic properties of muscular tissue^47^.

Five groups of tests, aiming to demonstrate the capability of the MFC to selectively steer different sets of magnets, were carried out. The tests differed in the stimulation frequency (low, 1 Hz, or high, 90 Hz) and in the type of variables being controlled, namely force ($\vec{F}$), torque ($\vec{\tau }$), or combinations of them. In other words, the tests differed in the number of DoFs under direct control (3, 6 or 12) and target magnets (from one to four). Each test lasted for 7 s. In all cases, only one magnet was selectively vibrated using a sinusoidal stimulus, either in a linear or torsional fashion, while the others were either kept still (under active control) or simply disregarded. In fact, it is recalled that with 12 coils no more than 12 DoFs could be physically controlled at a time. Only vibrations that could be observed and measured using the single video camera available, were tested (Fig. 2). Linear vibrations, or forces, were induced along two orthogonal axes (*x* and *y* in Fig. 2) perpendicular to the main axis of the cylindrical workspace. Except for PM4, these axes corresponded either to the magnetization axis, $\vec{m}$, or to an orthogonal axis of the magnet. For PM4, an additional linear vibration along its magnetization axis was tested, in specific tests (see hybrid control below). Torsional vibrations, or torques, were induced along the main axis of the workspace (*z* in Fig. 2). For PM3, however, this was not physically possible, being its magnetization axis parallel to *z*. Hence PM3 was (torsionally) vibrated along *x* and *y*.

#### Group I: control of $\vec{F}$ or $\vec{\tau }$ of one magnet (3-DoF control)

Each magnet was vibrated along the target directions using 3-DoF force control alone (for linear vibrations) or 3-DoF torque control alone (for torsional vibrations); the other DoFs (either 3-DoF torques or 3-DoF forces) were disregarded, as well as the other magnets. The test was repeated at low and high frequency, for a total of 28 trials (Supplementary Fig. S2, tests 1 to 28).

#### *Group II: control of*$\vec{F}$*and*$\vec{\tau }$*of one magnet (6-DoF control)*

Each magnet was vibrated along the target directions using 6-DoF force and torque control. The other magnets were disregarded. This test was repeated for all magnets at low and high frequency, for a total of 28 trials (Supplementary Fig. S3, tests 29 to 56).

#### Group III: control of $\vec{F}$ and $\vec{\tau }$ of two magnets (12-DoF control)

For each pair of magnets, each magnet was vibrated along the target directions using 6-DoF force and torque control, while the other magnet was kept fixed using 6-DoF force and torque control. The other two magnets were disregarded. This was repeated for all possible pairs, at low and high frequency, for a total of 45 trials (Supplementary Fig. S4, tests 57 to 101).

#### Group IV: control of $\vec{F}$ or $\vec{\tau }$ of four magnets, at low frequency (12-DoF control)

Each magnet was vibrated along the target directions using 3-DoF force control alone (for linear vibrations) or 3-DoF torque control alone (for torsional vibrations); the uncontrolled DoFs (either 3-DoF torques or 3-DoF forces) were disregarded. The other three magnets were kept fixed using either 3-DoF force control alone (when linear vibrations were tested) or 3-DoF torque control alone (when torsional vibrations were tested). Nine combinations were tested (Supplementary Fig. S5, tests 102 to 110).

#### Group V: hybrid control: control $\vec{F}$ and $\vec{\tau }$ of one magnet, and only $\vec{\tau }$ of other two magnets (12-DoF control)

Each magnet was vibrated along the target directions using 6-DoF force and torque control, while the other two magnets were kept fixed using 3-DoF torque control. PM1, PM2 and PM4 were controlled for this test. Actuation was performed at high frequency. A total of 8 tests were performed (Supplementary Fig. S5, tests 111 to 118).

Different amplitudes of $\vec{F}$ and $\vec{\tau }$ were required depending on the direction of the induced vibration. In particular, we ensured that $\vec{i}$, calculated using Eq. (12), never exceeded the current limits of the coils. In the cases where this was not possible, the test was skipped.

### Assessment of the remote vibrations: performance metrics

The actual movements of the magnets produced by the MFC were captured using one high-speed, high-resolution video camera (Sony DSC-RX10M4; 1000 fps max. frame rate, with a 1920 × 1080 max. resolution) which recorded a view on the *xy* plane (Fig. 2). The positions and orientations of the magnets were extracted from the video captures, using custom image processing algorithms (MATLAB, R2018b). The latter extracted the projection of the displacement along the desired axis (desired movement) and orthogonally (undesired movement), for linear vibrations on the *xy* plane. In the case of torsional vibrations, the only camera available allowed to extract the rotations of the magnets (in degrees) and their displacements along *x* and *y* (in mm), with respect to their neutral orientations and positions. The spectrum of each signal was extracted and its power spectral density was calculated to extract several parameters: (i) $P_{pos,d}$ and $P_{pos,d} |_{f}$, the power of the displacement signal along the desired actuation direction, in the whole spectrum and at the frequency of interest, respectively, when a force was applied, (ii) $P_{pos,u}$, the power of the displacement signal along the undesired (orthogonal) direction, in the whole frequency spectrum, (iii) $P_{ang}$ and $P_{ang} |_{f}$, the power of the displacement signal along the rotation direction, in the whole spectrum and at the frequency of interest, respectively, when a torque was applied.

For the tests involving linear vibrations (or forces) we then computed the following efficiency:

1 $$\eta = \frac{{P_{pos,d} |_{f} }}{{P_{pos,d} + P_{pos,u} }}$$

with one camera available, *η* accounted for the power losses due to movements in the *x* and *y* directions, but not for those in *z* (Fig. 2). Hence, *η* provided a compound information about the frequency selectivity and the directionality of the movement. A value of *η* close to one indicates thus a highly directional (i.e. applied along the desired direction) and frequency selective (i.e. with the signal power concentrated around the frequency of interest) vibration. In addition to *η*, we computed the torsional displacement signal, *θ(t)*, i.e. the undesired orientation of each magnet, for all relevant magnets. A distribution of *θ(t)* centered around zero with zero standard deviation (*σ*_*θ*_) meant pure linear movements.

In a similar way, for the tests involving torsional vibrations (or torques) we computed the following efficiency:

2 $$\gamma = \frac{{P_{ang} |_{f} }}{{P_{ang} }}.$$

Notably, due to the availability of one single camera, *γ* did not account for the power losses due to torques in the *x* and *y* directions (Fig. 2), but only along *z*. Hence, *γ* provided information about the frequency selectivity but not about the directionality. In analogy with the force control tests, here we also computed the linear displacement signal, *ρ(t)*, namely, the position vector of the center of mass, for all relevant magnets.

For the tests where multiple magnets were controlled by the MFC, we additionally computed the total linear displacement signal, *ρ*_*all*_*(t)*, and the total torsional displacement signal, *θ*_*all*_*(t)*. These signals were computed as the average of the individual *ρ(t)* and *θ(t)*, respectively, of all the magnets that were not supposed to move. Magnets that were not controlled by the MFC were not considered in the calculation. Distributions of *ρ*_*all*_*(t)* and *θ*_*all*_*(t)* around zero, with null standard deviation, meant selective vibrations (i.e. where only the desired magnet vibrates, and the others are kept fixed).

## Supplementary Information

Supplementary Information 1.

Supplementary Information 2.

## Data Availability

All data needed to evaluate the conclusions are available in the paper or the Supplementary Information. Please contact J.M. for other data and materials.
