# Supplementary material for: Feasibility of generating 90 Hz vibrations in remote implanted magnets
Source: Sci Rep. 2021 Jul 29;11:15456. doi: 10.1038/s41598-021-94240-2 (PMC8322332; doi:10.1038/s41598-021-94240-2)
Supplement: Supplementary file 2 — Supplementary Information 2. [file 41598_2021_94240_MOESM2_ESM.pdf]

Supplementary Information for

Feasibility of generating 90 Hz vibrations in remote implanted magnets

J. Montero, F. Clemente, C. Cipriani

Correspondence to: christian.cipriani@santannapisa.it

This PDF file includes:

Supplementary Text
Supplementary Figures S1 to S5

Other Supplementary Information for this manuscript include the following:

Movie S1

Supplementary Text

Equations modelling a myokinetic stimulation interface

Assuming that the far-condition is met, a permanent magnet or a coil can be approximated with the magnetic dipole model[1], which field can be described as:

$$\vec{B}_M = \frac{\mu_0}{4\pi}\left(\frac{3\vec{r}(\vec{m}\cdot\vec{r})}{\|\vec{r}\|^5} - \frac{\vec{m}}{\|\vec{r}\|^3}\right) \tag{3}$$

where $\vec{r}$ describes its position in a given reference frame and $\vec{m}$ corresponds to the magnetic moment vector. In particular, for a coil, $\vec{m}$ can be written as:

$$\vec{m}_c(t) = ki(t)\vec{l} \tag{4}$$

where k is a scaling factor, $i(t)$ is the current flowing through the coil, and $\vec{l}$ corresponds to the direction vector of the magnetic dipole. Equation (4) holds under the quasi-static approximation of the magnetic field[2], which indicates that the direction of the magnetic field vector can be approximated as that of a static field while its amplitude is modulated by the coil's current[2]. This approximation can be used in our case, considering that the frequencies required to stimulate the muscle spindles are relatively low (70-100 Hz range).

Using this, equation (3) can be rewritten as:

$$\vec{B}_{coil}\big(\vec{p}, i(t)\big) = \left[\frac{\mu_0 k}{4\pi}\left(\frac{3\vec{r}\big(\vec{l}\cdot\vec{r}\big)}{\|\vec{r}\|^5} - \frac{\vec{l}}{\|\vec{r}\|^3}\right)\right] i(t) = K(\vec{p})i(t) = \begin{bmatrix} k_x(\vec{p}) \\ k_y(\vec{p}) \\ k_z(\vec{p}) \end{bmatrix} i(t) \tag{5}$$

Notably, $K(\vec{p})$ represents the spatial distribution of the field, and depends only on the position. In turn, the amplitude of the magnetic field produced by the coil depends only on its current $i(t)$ (the time-index is dropped from here onwards for notational simplicity).

If C coils are used, and the current i_j of each one is independently controlled, then the compound magnetic field generated in a given point of space can be calculated as:

$$\begin{aligned} \vec{B}_{coils}(\vec{p}, \vec{\imath}) &= \sum_{j=1}^{C} K_j(\vec{p}) i_j = \begin{bmatrix} k_{x,1}(\vec{p}) \\ k_{y,1}(\vec{p}) \\ k_{z,1}(\vec{p}) \end{bmatrix} i_1 + \cdots + \begin{bmatrix} k_{x,C}(\vec{p}) \\ k_{y,C}(\vec{p}) \\ k_{z,C}(\vec{p}) \end{bmatrix} i_C \\ &= \begin{bmatrix} k_{x,1}(\vec{p}) & \cdots & k_{x,C}(\vec{p}) \\ k_{y,1}(\vec{p}) & \cdots & k_{y,C}(\vec{p}) \\ k_{z,1}(\vec{p}) & \cdots & k_{z,C}(\vec{p}) \end{bmatrix} \begin{bmatrix} i_1 \\ \vdots \\ i_C \end{bmatrix} = \overline{\overline{K}}(\vec{p})\vec{\imath} \end{aligned} \tag{6}$$

Where $\vec{\imath}$ (in $\mathbb{R}^C$) represents the vector of all coil currents, and matrix $\bar{\bar{K}}(\vec{p})$ (in $\mathbb{R}^{3\times C}$) maps the current vector to the 3D components of the magnetic field at point $\vec{p}$.

The torque developed by a permanent magnet (applied to its medium) can be calculated if the total magnetic field applied to it, $\vec{B}_T$, is known. Assuming that $\vec{B}_T$ is produced by C coils flown by current, and by close magnets, the torque can be expressed as[3]:

$$\vec{\tau} = \vec{m} \times \vec{B}_T = \vec{m} \times \left(\left[\sum_{j=1}^{C} \vec{B}_{coil,j} \right] + \vec{B}_D \right) = \left(\vec{m} \times \sum_{j=1}^{C} \begin{bmatrix} B_{x:coil,j} \\ B_{y:coil,j} \\ B_{z:coil,j} \end{bmatrix} \right) + \left(\vec{m} \times \vec{B}_D \right) = \vec{\tau}_{coils} + \vec{\tau}_D \qquad (7)$$

Where $\vec{B}_D$ is the disturbance field, generated by the other magnets, and $\vec{\tau}_D$ is its associated disturbance torque vector. Using equation (6), $\vec{\tau}_{coils}$ can be written as:

$$\begin{aligned} \vec{\tau}_{coils} &= \vec{m} \times \sum_{j=1}^{C} K_j(\vec{p}) i_j = \vec{m} \times \left(\begin{bmatrix} k_{x,1}(\vec{p}) \\ k_{y,1}(\vec{p}) \\ k_{z,1}(\vec{p}) \end{bmatrix} i_1 + \cdots + \begin{bmatrix} k_{x,C}(\vec{p}) \\ k_{y,C}(\vec{p}) \\ k_{z,C}(\vec{p}) \end{bmatrix} i_C \right) \\ &= \begin{bmatrix} \tau'_{x,1}(\vec{p},\vec{m}) & \cdots & \tau'_{x,C}(\vec{p},\vec{m}) \\ \tau'_{y,1}(\vec{p},\vec{m}) & \cdots & \tau'_{y,C}(\vec{p},\vec{m}) \\ \tau'_{z,1}(\vec{p},\vec{m}) & \cdots & \tau'_{z,C}(\vec{p},\vec{m}) \end{bmatrix} \vec{\imath} = \bar{\bar{K}}_\tau(\vec{p},\vec{m}) \vec{\imath} \end{aligned} \qquad (8)$$

Where matrix $\bar{\bar{K}}_\tau(\vec{p},\vec{m})$ (in $\mathbb{R}^{3\times C}$) maps the vector of coil currents to the 3D components of the torque. The entries of the matrix depend on the pose of the magnet with respect to each coil.

If N magnets are present in the workspace, then the torque induced by the C coils on each of them can be computed using equation (8). Doing so, it is possible to compute a matrix summarizing the effect of vector $\vec{\imath}$ over *all* the torques, as:

$$\vec{T} = \begin{bmatrix} \vec{\tau}_{coils,1} \\ \vdots \\ \vec{\tau}_{coils,N} \end{bmatrix} = \begin{bmatrix} \tau'_{x1,1}(\vec{p}_1,\vec{m}_1) & \cdots & \tau'_{x1,C}(\vec{p}_1,\vec{m}_1) \\ \tau'_{y1,1}(\vec{p}_1,\vec{m}_1) & \cdots & \tau'_{y1,C}(\vec{p}_1,\vec{m}_1) \\ \tau'_{z1,1}(\vec{p}_1,\vec{m}_1) & \cdots & \tau'_{z1,C}(\vec{p}_1,\vec{m}_1) \\ \vdots & \vdots & \vdots \\ \tau'_{xN,1}(\vec{p}_N,\vec{m}_N) & \cdots & \tau'_{xN,C}(\vec{p}_N,\vec{m}_N) \\ \tau'_{yN,1}(\vec{p}_N,\vec{m}_N) & \cdots & \tau'_{yN,C}(\vec{p}_N,\vec{m}_N) \\ \tau'_{zN,1}(\vec{p}_N,\vec{m}_N) & \cdots & \tau'_{zN,C}(\vec{p}_N,\vec{m}_N) \end{bmatrix} \vec{\imath} = \bar{\bar{M}}_\tau \vec{\imath} \qquad (9)$$

Where matrix $\bar{\bar{M}}_\tau$ describes the torque generated by vector $\vec{\imath}$ on all magnets. Its dimensions are $3N \times C$ (i.e., $\mathbb{R}^{3N\times C}$). Three coils are thus required per magnet, in order to control its full torque vector. Using fewer coils leads to an under-actuated system. Using more than 3*N* coils leads to an over-actuated system. Matrix $\bar{\bar{M}}_\tau$ is always rank deficient, thus meaning that some torque configurations (vectors) are unfeasible. This is due to the fact that a magnet cannot rotate around its central axis, under the influence of an external magnetic field.

The force exerted by C coils on a magnet can be computed using a procedure analogous to the previously described one. The total force can be decomposed into a disturbance force $\vec{f}_D$, generated by nearby magnets, and a coil-generated force $\vec{f}_{coils}$. If the magnetic dipole model is used to describe the coils magnetic field, then $\vec{f}_{coils}$ can be computed as[4]:

$$\begin{aligned}\vec{f}_{coils} &= \frac{3\mu_0}{4\pi}\sum_{j=1}^{C}\frac{1}{\|\vec{r}_j\|^5}\left[(\vec{m}\cdot\vec{r}_j)\vec{m}_j + (\vec{m}_j\cdot\vec{r}_j)\vec{m} + (\vec{m}_j\cdot\vec{m})\vec{r}_j - \frac{5(\vec{m}_j\cdot\vec{r}_j)(\vec{m}\cdot\vec{r}_j)\vec{r}_j}{\|\vec{r}_j\|^2}\right] \\ &= \begin{bmatrix} f'_{x,1}(\vec{p},\vec{m}) & \cdots & f'_{x,C}(\vec{p},\vec{m}) \\ f'_{y,1}(\vec{p},\vec{m}) & \cdots & f'_{y,C}(\vec{p},\vec{m}) \\ f'_{z,1}(\vec{p},\vec{m}) & \cdots & f'_{z,C}(\vec{p},\vec{m}) \end{bmatrix}\vec{\imath} = \bar{\bar{K}}_f(\vec{p},\vec{m})\vec{\imath}\end{aligned} \tag{10}$$

By using this equation to compute the force that each coil exerts over each magnet, it is possible to construct matrix $\bar{\bar{M}}_f$ as:

$$\vec{F} = \begin{bmatrix} \vec{f}_{coils,1} \\ \vdots \\ \vec{f}_{coils,N} \end{bmatrix} = \begin{bmatrix} f'_{x1,1}(\vec{p}_1,\vec{m}_1) & \cdots & f'_{x1,C}(\vec{p}_1,\vec{m}_1) \\ f'_{y1,1}(\vec{p}_1,\vec{m}_1) & \cdots & f'_{y1,C}(\vec{p}_1,\vec{m}_1) \\ f'_{z1,1}(\vec{p}_1,\vec{m}_1) & \cdots & f'_{z1,C}(\vec{p}_1,\vec{m}_1) \\ \vdots & \vdots & \vdots \\ f'_{xN,1}(\vec{p}_N,\vec{m}_N) & \cdots & f'_{xN,C}(\vec{p}_N,\vec{m}_N) \\ f'_{yN,1}(\vec{p}_N,\vec{m}_N) & \cdots & f'_{yN,C}(\vec{p}_N,\vec{m}_N) \\ f'_{zN,1}(\vec{p}_N,\vec{m}_N) & \cdots & f'_{zN,C}(\vec{p}_N,\vec{m}_N) \end{bmatrix}\vec{\imath} = \bar{\bar{M}}_f\vec{\imath} \tag{11}$$

Matrix $\bar{\bar{M}}_f$ ($\in \mathbb{R}^{3N\times C}$) describes the mapping from the currents in the coils to the forces of the magnets. Three coils per magnet are required to independently control each force vector.

Using $\bar{\bar{M}}_\tau$ and $\bar{\bar{M}}_f$, it is possible to calculate the control matrix $\bar{\bar{M}}$. This matrix is required by the system to calculate the currents necessary to achieve a desired force/torque configuration. The matrix can be constructed in different ways: i) $\bar{\bar{M}}_\tau$ and $\bar{\bar{M}}_f$ can be stacked together, if the aim is to control the force and the torque of each magnet; ii) $\bar{\bar{M}}$ can be constructed using only the rows of $\bar{\bar{M}}_f$, or those of $\bar{\bar{M}}_\tau$, in which case only the forces or the torques are controlled, respectively; iii) rows can be arbitrarily mixed together; in such case, e.g. the forces and torques of one magnet might be fully controlled, while only the forces or the torques of the remaining magnets are controlled.

Case i) requires the use of at least $6N$ coils; this is required if, for instance, it is desired to control their motion across the workspace, as in the works by Diller and colleagues[5] and Chowdhury and team[6]. Case ii) requires at least $3N$ coils, given that only one of the vectors (forces or torques) is controlled. This can be used if, for instance, the magnet is constrained by the medium and cannot move in a given direction. Case iii) requires as many coils as variables being controlled.

Thus by unifying equations (9) and (11) to construct $\bar{\bar{M}}$, and using its inverse (or the pseudoinverse), and the vector of reference signals $\vec{r}$ (forces and/or torques), it is possible to compute the required currents as:

$$\vec{\imath} = \bar{\bar{M}}^{-1}\left(\vec{r} - \vec{d}\right) \tag{12}$$

Where $\vec{d}$ includes the disturbance terms due to the nearby magnets. Its entries are extracted from $\vec{\tau}_D$ and $\vec{f}_D$, depending on how matrix $\bar{\bar{M}}$ is constructed. Vector $\vec{d}$ can be neglected, if its entries are negligible with respect to those of $\vec{r}$.

References

[1] Yang, W., Hu, C., Li, M., Meng, M. Q. H. & Song, S. A new tracking system for three magnetic objectives. *IEEE transactions on magnetics*. **46**, 4023-4029 (2010).

[2] Furse, C., Christensen, D. A. & Durney, C. H. *Basic Introduction to Bioelectromagnetics* (ed. 2). (CRC Press, Florida, 2009).

[3] Sadiku, M. *Elements of Electromagnetics* (ed. 3). (Oxford University Press, 2000).

[4] Yung, K. W., Landecker, P. B. & Villani, D. D. An analytic solution for the force between two magnetic dipoles. *Magnetic and Electrical Separation*. **9**, 39-52 (1998).

[5] Diller, E., Giltinan, J. & Sitti, M. Independent control of multiple magnetic microrobots in three dimensions. *The International Journal of Robotics Research*. **32**, 614-631 (2013).

[6] Chowdhury, S., Jing, W. & Cappelleri, D. J. Towards independent control of multiple magnetic mobile microrobots. *Micromachines*. **7** (2016).

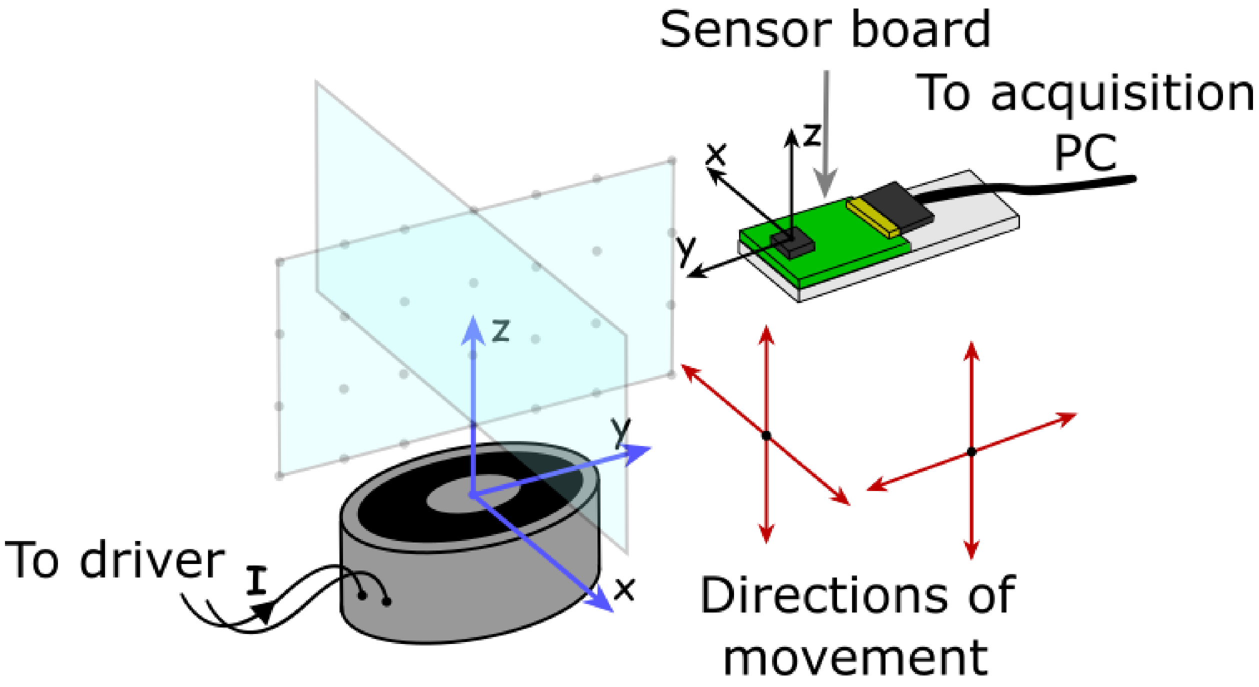

Supplementary Figure S1.

Setup for the characterization of the electromagnetic coils. The magnetic field generated by the coil was sampled along two orthogonal, cross-shaped planes using a sensor mounted on a positioning platform. Symmetry was exploited to derive a 3D model. The process was repeated for different input current amplitudes and the average of the model parameters obtained for the different currents was calculated.

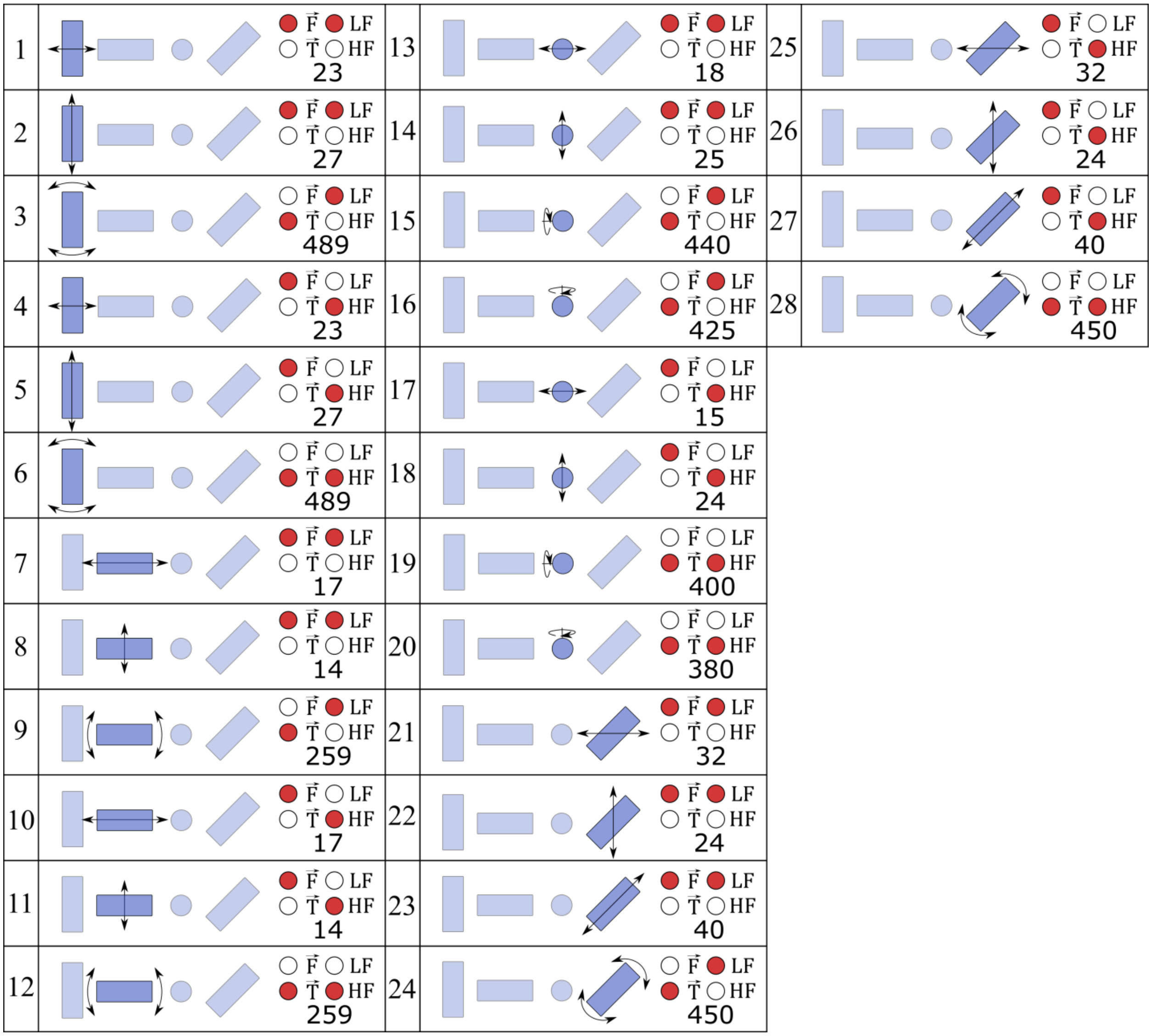

Supplementary Figure S2.

Test conditions for Group I (3-DoF control). The 3-DoF control tests consisted in generating linear vibrations, through force ($\vec{F}$) control, or torsional vibrations, through torque ($\vec{T}$) control, in a single magnet. Tests were performed at 1 Hz (*LF*) and at 90 Hz (*HF*). The number specified under the test conditions corresponds to the amplitude of the sinusoidal reference signal (in mN for force signals and μNm for torque signals).

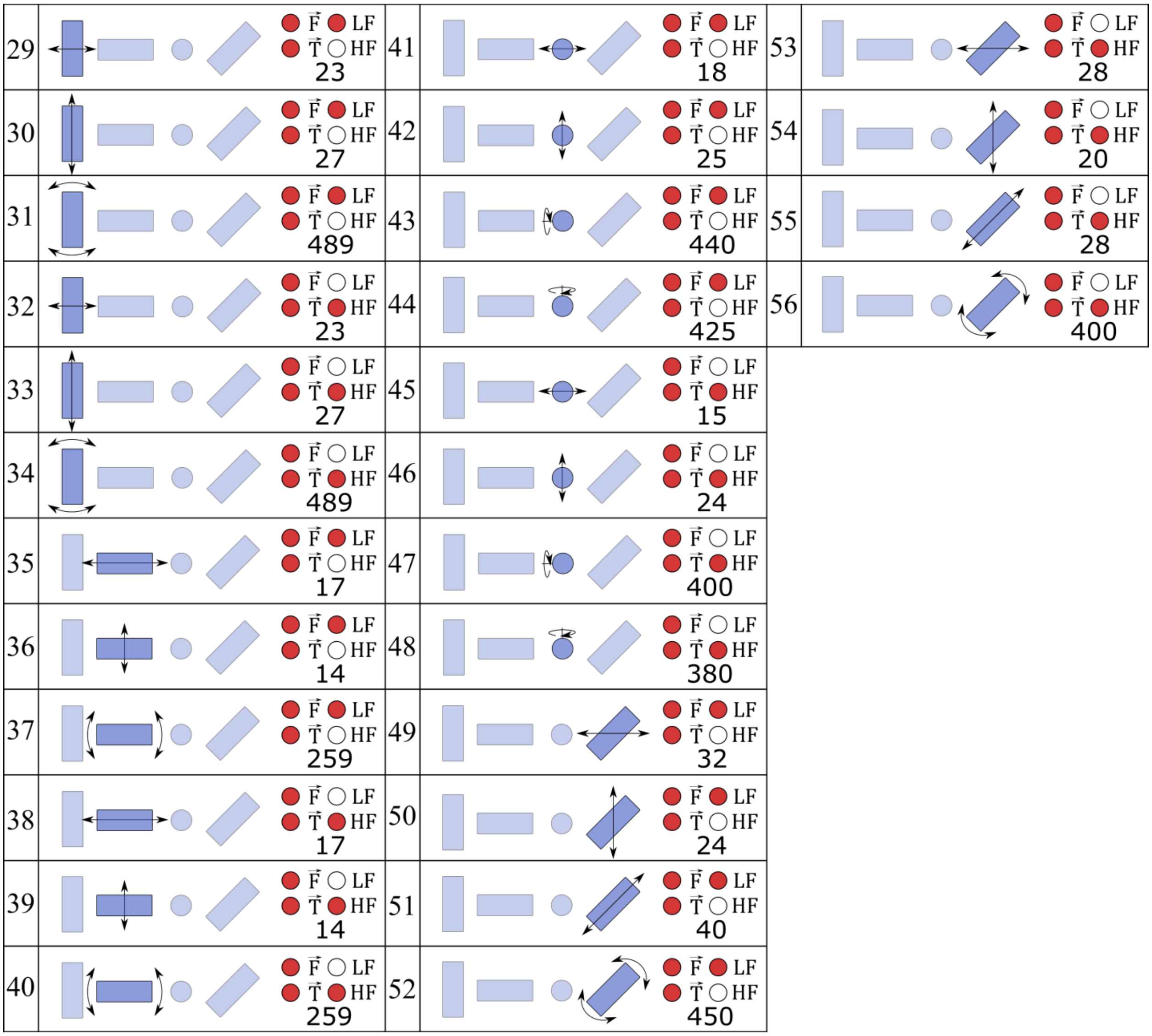

Supplementary Figure S3.

Test conditions for Group II (6-DoF control). The 6-DoF control tests consisted in generating linear or torsional vibrations in a single magnet. During these tests, the forces *and* the torques of the magnet were controlled. Tests were performed at 1 Hz (*LF*) and at 90 Hz (*HF*). The number specified under the test conditions corresponds to the amplitude of the sinusoidal reference signal (in mN for force signals and μNm for torque signals).

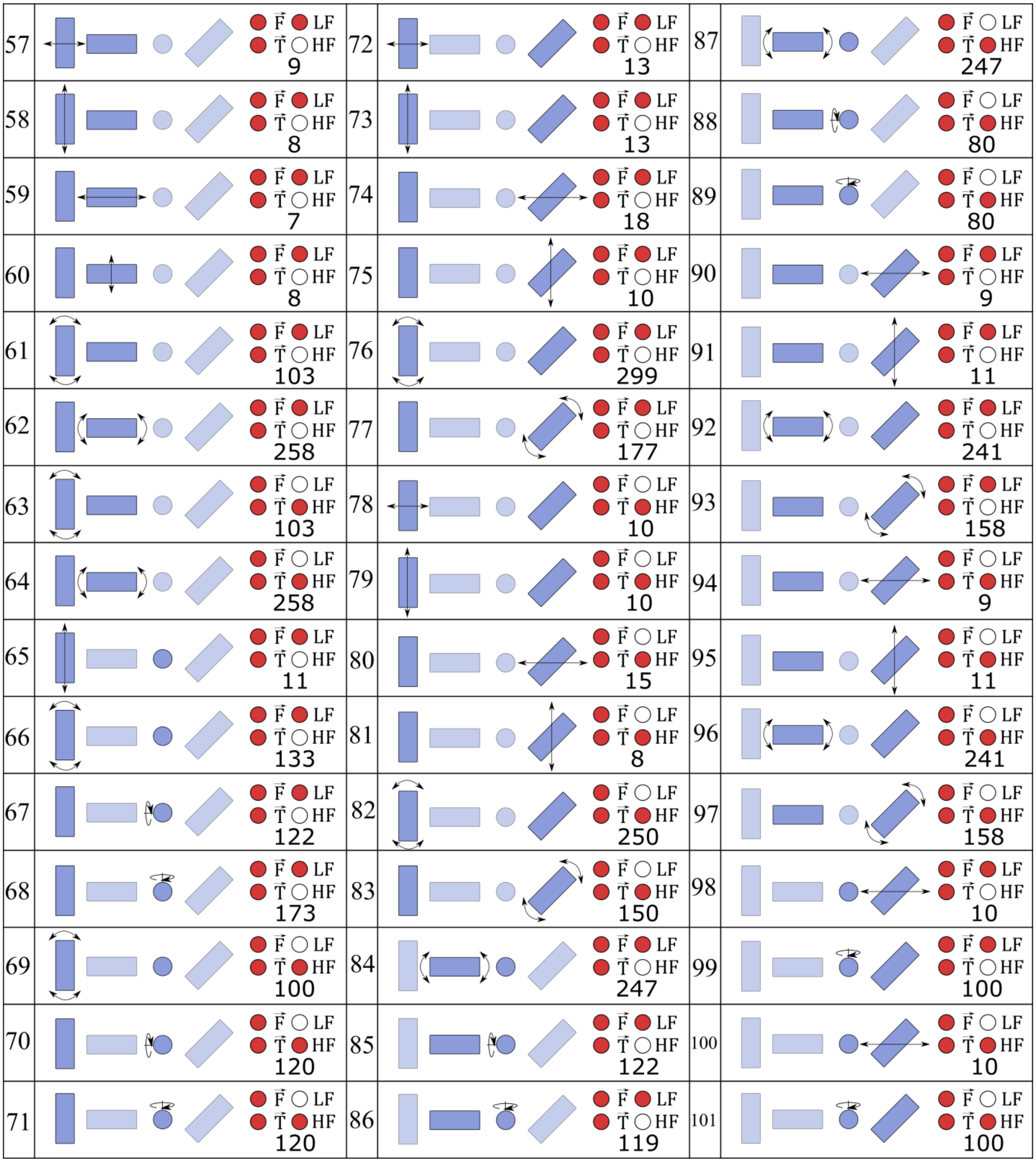

Supplementary Figure S4.

Test conditions for Group III (2 magnets, 12-DoF control). The two-magnet, 12-DoF control tests consisted in generating linear or torsional vibrations in a single magnet (indicated with the arrows), while keeping fixed a second one (with no arrows). The remaining magnets (dimmed) were disregarded by the MFC. During these tests, the forces *and* the torques of the two magnets were controlled. Tests were performed at 1 Hz (*LF*) and at 90 Hz (*HF*). The number specified

under the test conditions corresponds to the amplitude of the sinusoidal reference signal (in mN for force signals and μNm for torque signals).

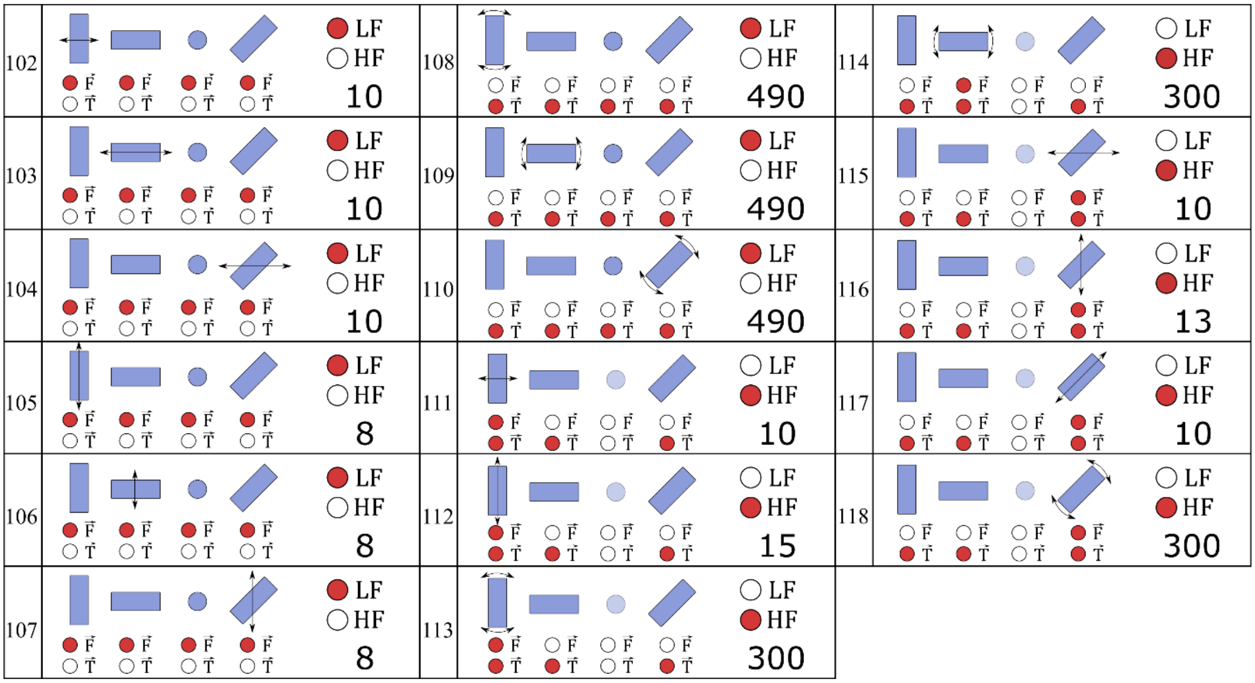

Supplementary Figure S5.

Test conditions for Groups IV and V (3 or 4 magnets, 12-DoF control). The four-magnet, 12-DoF control tests (Group IV, tests number 102 to 110) consisted in generating linear or torsional vibrations in a single magnet (indicated with the arrows), while keeping fixed all others. During these tests, only the forces *or* the torques of each magnet were controlled. The three-magnet, 12-DoF control tests (Group V – Hybrid control, tests number 111 to 118) consisted in generating linear or torsional vibrations in a single magnet (indicated with the arrows), while keeping two other magnets (highlighted, no arrows). During these tests, the forces *and* the torques of the vibrated magnet were controlled, and only the forces (for linear vibrations) or the torques (for torsional vibrations) of the other two were controlled. Tests were performed at 1 Hz (*LF*) and at 90 Hz (*HF*). The number specified under the test conditions corresponds to the amplitude of the sinusoidal reference signal (in mN for force signals and μNm for torque signals). Dimmed magnets were disregarded by the MFC.
